# Vaccine Based on Recombinant Fusion Protein Combining Hepatitis B Virus PreS with SARS-CoV-2 Wild-Type- and Omicron-Derived Receptor Binding Domain Strongly Induces Omicron-Neutralizing Antibodies in a Murine Model

**DOI:** 10.3390/vaccines12030229

**Published:** 2024-02-23

**Authors:** Pia Gattinger, Bernhard Kratzer, Al Nasar Ahmed Sehgal, Anna Ohradanova-Repic, Laura Gebetsberger, Gabor Tajti, Margarete Focke-Tejkl, Mirjam Schaar, Verena Fuhrmann, Lukas Petrowitsch, Walter Keller, Sandra Högler, Hannes Stockinger, Winfried F. Pickl, Rudolf Valenta

**Affiliations:** 1 Division of Immunopathology, Department of Pathophysiology and Allergy Research, Infectiology and Immunology, Center for Pathophysiology, Medical University of Vienna, 1090 Vienna, Austria; 2 Institute of Immunology, Infectiology and Immunology, Center for Pathophysiology, Medical University of Vienna, 1090 Vienna, Austria; 3 Institute for Hygiene and Applied Immunology, Infectiology and Immunology, Center for Pathophysiology, Medical University of Vienna, 1090 Vienna, Austrialaura.gebetsberger@meduniwien.ac.at (L.G.);; 4 Karl Landsteiner University of Health Sciences, 3500 Krems, Austria; 5 Institute of Molecular Biosciences, BioTechMed Graz, University of Graz, 8010 Graz, Austria; 6 Unit of Laboratory Animal Pathology, Institute of Pathology, University of Veterinary Medicine Vienna, 1210 Vienna, Austria; 7 Laboratory for Immunopathology, Department of Clinical Immunology and Allergology, Sechenov First Moscow State Medical University, 119991 Moscow, Russia; 8 NRC Institute of Immunology FMBA of Russia, 115478 Moscow, Russia

**Keywords:** SARS-CoV-2, COVID-19, Omicron, vaccine, neutralizing antibodies

## Abstract

Background: COVID-19, caused by the severe acute respiratory syndrome coronavirus 2 (SARS-CoV-2), has become a recurrent endemic disease affecting the whole world. Since November 2021, Omicron and its subvariants have dominated in the spread of the disease. In order to prevent severe courses of disease, vaccines are needed to boost and maintain antibody levels capable of neutralizing Omicron. Recently, we produced and characterized a SARS-CoV-2 vaccine based on a recombinant fusion protein consisting of hepatitis B virus (HBV)-derived PreS and two SARS-CoV-2 wild-type RBDs. Objectives: To develop a PreS-RBD vaccine which induces high levels of Omicron-specific neutralizing antibodies. Methods: We designed, produced, characterized and compared strain-specific (wild-type: W-PreS-W; Omicron: O-PreS-O), bivalent (mix of W-PreS-W and O-PreS-O) and chimeric (i.e., W-PreS-O) SARS-CoV-2 protein subunit vaccines. Immunogens were characterized in vitro using protein chemical methods, mass spectrometry, and circular dichroism in combination with thermal denaturation and immunological methods. In addition, BALB/c mice were immunized with aluminum–hydroxide-adsorbed proteins and aluminum hydroxide alone (i.e., placebo) to study the specific antibody and cytokine responses, safety and Omicron neutralization. Results: Defined and pure immunogens could be produced in significant quantities as secreted and folded proteins in mammalian cells. The antibodies induced after vaccination with different doses of strain-specific, bivalent and chimeric PreS-RBD fusion proteins reacted with wild-type and Omicron RBD in a dose-dependent manner and resulted in a mixed Th1/Th2 immune response. Interestingly, the RBD-specific IgG levels induced with the different vaccines were comparable, but the W-PreS-O-induced virus neutralization titers against Omicron (median VNT50: 5000) were seven- and twofold higher than the W-PreS-W- and O-PreS-O-specific ones, respectively, and they were six-fold higher than those of the bivalent vaccine. Conclusion: Among the tested immunogens, the chimeric PreS-RBD subunit vaccine, W-PreS-O, induced the highest neutralizing antibody titers against Omicron. Thus, W-PreS-O seems to be a highly promising COVID-19 vaccine candidate for further preclinical and clinical evaluation.

## 1. Introduction

The COVID-19 pandemic, which broke out in late 2019, has been responsible for several million fatalities and multifaceted disease-associated chronic illnesses [1,2,3,4]. Since then, the sequence, structure and host cell entry mechanisms of SARS-CoV-2, as well as the innate and adaptive immune responses after infection, have been studied in great detail [5,6,7]. The binding of SARS-CoV-2 via its receptor-binding domain (RBD) to its cognate receptor ACE2 on human cells has been revealed as a critical target for active and passive immunization strategies and anti-viral treatment regimens [8,9,10]. Accordingly, treatments focusing on the ACE2–RBD interaction can be studied using virus-neutralization tests and molecular interaction assays (MIAs) [11,12].

During the initial evolution of SARS-CoV-2 from the original strain toward other variants (e.g., Alpha to Delta), the sequence and structure of the RBD had remained highly conserved so that vaccines and therapeutic antibodies developed against the original strain retained their effectiveness [13,14,15].

However, at the end of 2021, a novel variant, termed Omicron, emerged, which differed substantially from all previous variants in the sequence of the spike protein S and especially in its RBD [15,16]. It transpired that the available vaccines and therapeutic antibodies showed a reduced efficacy for Omicron [13,15]. Although Omicron seemed to cause milder forms of COVID-19 in the general population [17,18], the decreased effects of the available active and passive immunizations [13,15,19,20] became a major concern, especially for vulnerable persons. In particular, elderly subjects [21], patients suffering from malignant diseases under therapy, immunocompromised patients [22] and patients with immunodeficiencies [23] showed a strongly reduced adaptive immunity to Omicron and continued to be at risk of developing severe COVID-19.

We previously found that only vaccines including structurally preserved and folded RBD, but not unfolded RBD, can induce SARS-CoV-2-neutralizing antibody responses [24]. Based on this knowledge, we generated a SARS-CoV-2 vaccine based on two RBDs from the original Wuhan-hu-1 (wild-type) strain fused to the hepatitis B virus PreS antigen [25]. This vaccine antigen, termed PreS-RBD, was expressed as a recombinant folded fusion protein and, upon immunization, induced a potent neutralizing antibody response against the SARS-CoV-2 wild-type strain. PreS-RBD-induced antibodies reacted not only with wild-type RBD but also showed strong cross-reactivity with a variety of SARS-CoV-2 variants, including Omicron [25].

The goal of this study was to refine the PreS-RBD vaccine for Omicron. For this purpose, we developed and compared two subunit vaccines which are strain-specific (Wuhan hu-1 wild-type: W-PreS-W; Omicron: O-PreS-O), a bivalent vaccine based on a mix of W-PreS-W and O-PreS-O and a chimeric vaccine combining RBDs from Wuhan hu-1 wild-type and Omicron in a single fusion protein (W-PreS-O).

Here, we report on the biochemical and biophysical characterization of the vaccine antigens, the comparison of their immunogenicity in a murine model and their abilities to induce Omicron-neutralizing antibodies via virus neutralization.

## 2. Materials and Methods

### 2.1. Expression and Purification of Recombinant Proteins

**Figure 1:**
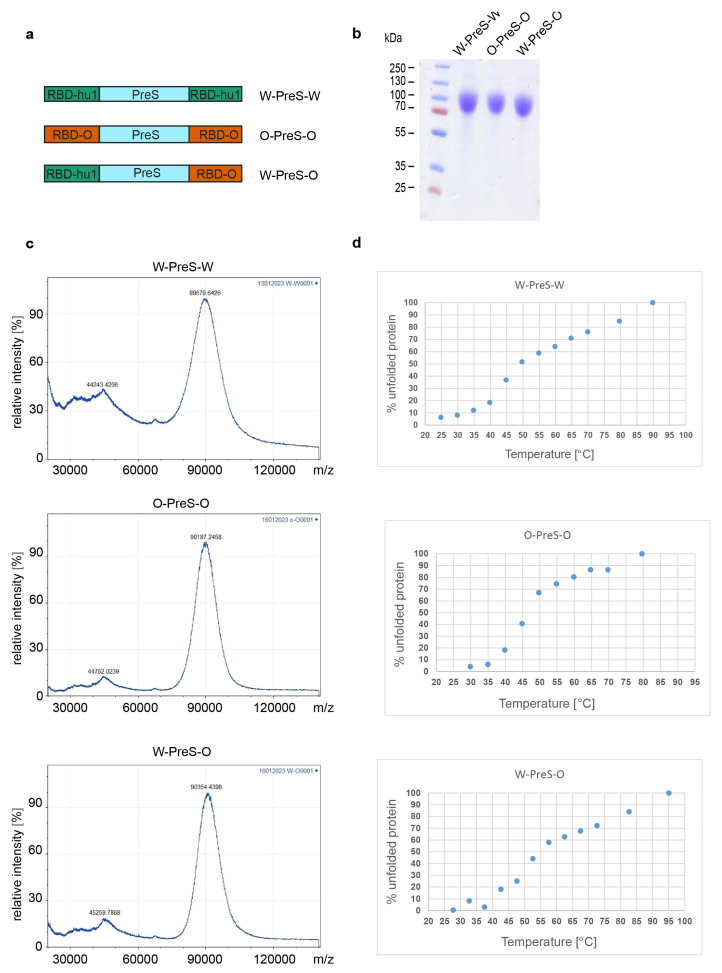
(**a**) Scheme of the recombinant PreS-RBD fusion proteins. RBD domains from hu-1 (green) and from Omicron (orange) were fused to the N- and C-terminus of PreS (blue) as indicated. (**b**) Coomassie blue-stained SDS-PAGE of HEK cell-expressed and purified PreS-RBD fusion proteins. Molecular weights are indicated in kDa on the left margin. (**c**) MALDI analyses of fusion proteins. Y-axes: relative intensity as percentage of most abundant signal intensity. X-axes: mass/charge ratio. (**d**) Melting curve of PreS-RBD fusion proteins. The melting curves are shown forPreS-RBD fusion proteins as percent unfolded protein (y-axes) at increasing temperatures in °C (x-axes).

Three recombinant PreS-RBD fusions were based on synthetic genes containing a cDNA coding for HBV-derived PreS, which was flanked at the 5′ and 3′ end via DNA sequences coding for a N-terminal and C-terminal SARS-CoV-2 RBD. The synthetic genes were codon optimized for HEK293 cell expression; they contained a 5′ DNA coding for an IL-2 signal peptide and a 3′ DNA coding for a hexahistidine tag and were cloned into the BamHI and EcoRI sites of plasmid pcDNA3.1(+) (GenScript, Leiden, The Netherlands). The expression in the HEK293F cells and the subsequent purification via Ni-NTA agarose were performed as previously described [25]. Figure 1a provides an overview of the corresponding recombinant fusion proteins. For the W-PreS-W fusion, the amino acid sequences of RBD derived from the SARS-CoV-2 wild-type strain hu-1 (GenBank accession Nr.: QHD43416.1) were used as previously described [25]. For O-PreS-O, the RBD-encoding sequence from the SARS-CoV-2 Omicron BA.1 (Pango B.1.1.529) was used and for W-PreS-O, the PreS was flanked with an N-terminal RBD-hu-1 and a C-terminal RBD from Omicron BA.1. Recombinant proteins were analyzed for purity using SDS-PAGE and Coomassie blue staining (Figure 1b).

### 2.2. Matrix-Assisted Laser Desorption and Ionization Time-of-Flight Mass Spectrometry

Laser desorption mass spectra of the recombinant proteins were acquired with an Axima Confidence matrix-assisted laser desorption and ionization instrument (Shimadzu Biotech, Kyoto, Japan). Purified PreS-RBD fusion proteins were mixed 1:1 with saturated sinapinic acid as the matrix and applied to the target (Kratos Analytical, Manchester, UK) using the pre-coated dried droplet technique. The measurements were performed in linear mode with a laser power of 120. The calibration was performed with standard proteins (cytochrome C, carboxyanhydrase, BSA, all Sigma-Aldrich, St. Louis, MO, USA). The results are given as the relative intensity to mass-to-charge ratio (*m*/*z*) and were obtained with mMass software [26] (Open Source Mass Spectrometry Tool: http://www.mmass.org/ (accessed on 1 December 2023)) (Figure 1c).

### 2.3. Thermal Denaturation, Renaturation and Determination of the Melting Temperature via Circular Dichroism (CD)

The far UV circular dichroism (CD) spectra of the PreS-fusion proteins were collected on a Jasco J-1500 CD Spectrometer (Japan Spectroscopic Co., Tokyo, Japan) using a 1 mm path length quartz cuvette at protein concentrations of 0.5 mg/ml. The spectra were measured from 260 to 190 nm and recorded through an increase in the temperature from 20 to 95 °C at a heating rate of 1 °C/min. The results were expressed as the mean residue ellipticity (θ) at a given wavelength. At 230 nm, the % of folded and unfolded protein was calculated with θ at 20 °C as 0% unfolded and θ at 95 °C as 100% unfolded. The melting temperature was determined by non-linear least-squares data fitting (Figure 1d) [27].

### 2.4. Immunization of Mice, Blood Sampling and Manipulations 

**Figure 2:**
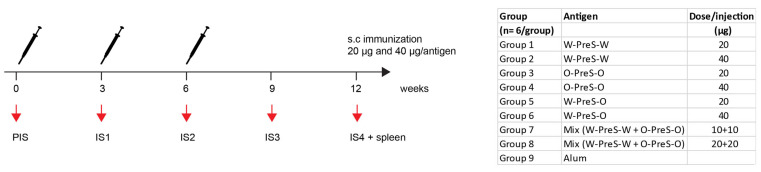
Scheme of immunizations and treatments. Mice (*n* = 6/group) were immunized subcutaneously three times at three-weekly intervals with 20 µg or 40 µg of PreS-RBD fusion proteins or alum as control. Time points of blood sampling (PIS, IS1, IS2, IS3, IS4) and splenectomy (IS4) are indicated.

BALB/c mice (female, age: 6 to 8 weeks) were purchased from Charles River (Kisslegg, Germany) and the experimental procedures were approved by the Animal Ethics Committee of the Medical University of Vienna and the Austrian Federal Ministry of Science, Research and Economy (2022-0.301.523). Groups of mice (*n* = 6) were immunized subcutaneously three times at three-weekly intervals with 20 µg or 40 µg PreS-RBD fusion proteins (Figure 2) adsorbed to aluminum hydroxide (Alu-Gel-S; SERVA, Heidelberg, Germany) with a final volume of 150 µl (0.39 mg aluminum hydroxide/ml, 10 mM NaH_2_PO_4_, 0.9% NaCl, pH 7.2). The serum samples were obtained from tail veins before the first immunization (PIS) and before the second and third immunization (IS1, IS2) as well as 3 weeks (IS3) and 6 weeks (IS4) after the third immunization (Figure 2). Thereafter, the mice were sacrificed and spleens were removed. For six mice immunized with 40 µg PreS-RBD fusion protein mix (Figure 2), and three non-immunized control mice, histopathological examinations were performed.

### 2.5. Measurements of Specific Antibodies 

IgG_1_ and IgG_2a_ antibody levels specific for HEK cell-expressed RBD-hu-1 [25] and RBD-Omicron (BA.1) (GenScript) were measured using ELISA in mouse serum samples. Recombinant proteins were coated overnight (2 µg/ml) at 4 °C onto NUNC Maxisorp 96-well plates (ThermoFisher, Waltham, MA, USA). After blocking with 2% BSA/PBST, the mouse serum samples were added (1:500 to 1:8000 dilutions as indicated in the figure legends) and incubated overnight at 4 °C. After washing three times with PBST, 1:1000 diluted purified rat anti-mouse IgG_1_ or IgG_2a_ (both BD Pharmingen, NJ, USA) were added and incubated for two hours. Afterwards, plates were washed three times and incubated for one hour with 1:2000 diluted HRP-labeled goat anti-rat antibodies (GE Healthcare UK Limited, Chalfont St Giles, United Kingdom). Bound antibodies were detected with ABTS, and optical density (OD) was measured at 405/492 nm with the Infinite F50 ELISA reader after 10 min (Tecan, Männedorf, Switzerland). In order to allow a comparison of OD levels, reference sera were included on each plate for standardization. Thus, semi-quantitative OD levels obtained in the experiments can be directly compared. All measurements were performed in duplicate with a <5% difference, and results are given as averages of duplicates.

### 2.6. Virus Neutralization Assays

The neutralization of SARS-CoV-2 Omicron BA.1 was determined by measuring 50% virus neutralization titers of mouse serum samples obtained six weeks after three immunizations (IS4) as previously described [25,28]. Triplicate determinations for each serum sample (*n* = 6 per group, except for W-PreS-W 20 µg, due to lack of sera *n* = 5) were performed. The 50% virus neutralization titer (VNT50) was reported as the interpolated reciprocal of the dilution, yielding a 50% reduction in the anti-SARS-CoV-2 nucleocapsid protein staining.

### 2.7. Measurement of Specific Cytokine Production 

The spleens of immunized mice were removed under aseptic conditions six weeks after the third immunization (Figure 2), and the splenocytes were isolated and stimulated with 5 µg/mL RBD-hu-1, RBD-Omicron BA.1 or medium for 5 days as previously described [29]. Thereafter, supernatants were analyzed regarding their specific mouse IL-2, IL-4, IL-5, IL-10, IL-12(p70), GM-CSF, IFN-γ and TNF-α concentrations with the Bio-Plex Pro Mouse Cytokine Th1/Th2 panel (Bio-Rad Laboratories, CA, USA) following the manufacturer’s instructions. All cellular stimulations and cytokine measurements were performed in triplicates and calculated as average values for each individual mouse. Specific cytokine production upon stimulation with antigens is shown as the stimulation index and was calculated as the average measurement of cytokines divided by the average of triplicate of unstimulated cells.

### 2.8. Histological Examination

A full necropsy was performed for nine animals (highest dose, i.e., 40 µg mix *n* = 6; no treatment *n* = 3) and samples of liver, spleen, kidneys, lungs and heart were fixed in 4% buffered formalin and embedded in paraffin for histological evaluation. A pathologist blinded to the group assignments provided evaluations in a descriptive manner of the hematoxylin–eosin-stained sections.

### 2.9. Statistics

For the group size calculations of mice, the GINGER tool Version 1.2 of the Institute of Clinical Biometry, Medical University of Vienna [30] was used. With a two-sided significance level of 0.05, the pairwise post hoc comparisons using Tukey’s HSD correction between 9 groups with 6 subjects in each group have 80% power to detect a mean difference in the primary outcome variable IgG of 0.5 OD if the within-group standard deviation is 0.2 OD, corresponding to an effect size of 2.5.

The differences in immunoglobulin reactivity and VNT50 titers and specific cytokines shown as the stimulation index were determined using a two-tailed Mann–Whitney U-test with a 95% confidence interval, and correlations were assessed using Spearman’s rank correlation with GraphPad Prism Version 5.00 (La Jolla, CA, USA). *p* values of <0.05 were considered to be significant.

## 3. Results

### 3.1. Recombinant Vaccine Antigens Represent Defined, Folded and Stable Proteins 

For the last two years, Omicron and its subvariants have been the major cause of COVID-19. Accordingly, it was our goal to refine our previously described subunit vaccine, which was based on a recombinant PreS-RBD fusion protein containing two SARS-CoV-2 wild-type RBDs for vaccination against Omicron. For this purpose, we compared the earlier described wild-type-derived (W-PreS-W) with a new construct containing two RBDs from Omicron (O-PreS-O) and a chimeric “all-in-one” protein with one RBD from wild type and another from Omicron (W-PreS-O) (Figure 1a). The three proteins were expressed in HEK cells and subsequently purified to homogeneity using nickel affinity chromatography. The three glycoproteins were visualized as bands of approximately 90 kDa via SDS-PAGE (Figure 1b). Mass spectrometry revealed prominent peaks at 89,678 Da, 90,187 Da and 90,354 Da for W-PreS-W, O-PreS-O and W-PreS-O, respectively (Figure 1c). The smaller peaks at 44,243 Da (W-PreS-W), 44,752 Da (O-PreS-O) and 45,259 Da (W-PreS-O) corresponded to the doubly charged forms of the proteins (Figure 1c). Thus, the results obtained for the three recombinant glycoproteins via SDS-PAGE and mass spectrometry are in good agreement. Since it has previously been reported that the ability of the RBD and PreS-RBD proteins to induce neutralizing antibodies depends on the intact fold of the respective proteins, we were interested in examining the thermal stability of the three antigens. For this purpose, the percentage of folded protein was analyzed from +20 °C to nearly +100 °C using circular dichroism spectroscopy (Figure 1d). The recombinant proteins seemed to be quite temperature stable with melting points for W-PreS-W of 53.1 °C, for O-PreS-O of 44.9 °C and for W-PreS-O of 57.2 °C (Figure 1d). More than 80% of each of the proteins remained folded up to a temperature of 40 °C.

### 3.2. Formulation of the Subunit Vaccines and Immunization Schedule

We previously developed allergen-specific immunotherapy vaccines based on fusion proteins consisting of PreS and allergen-derived peptides which were formulated by adsorption to aluminum hydroxide [31]. These vaccines were shown to be safe in clinical immunotherapy trials and induced antibodies blocking the allergen–IgE interaction. Accordingly, we formulated PreS-RBD fusion protein vaccines through adsorbing different amounts (20 μg, 40 μg) of PreS-RBD fusion proteins (W-PreS-W, O-PreS-O, W-PreS-O) or equimolar mixes of them (W-PreS-W + O-PreS-O; i.e., 10 + 10 μg or 20 + 20 μg) (Figure 2). Aluminum hydroxide alone was used as the negative control. Three subcutaneous immunizations were performed at three-weekly intervals (Figure 2). For a preliminary analysis of safety, the parameters of body weight gain, clinical symptoms, full necropsy and histopathological examination were assessed. No significant differences in weight and weight gain between groups immunized with PreS-RBD fusion constructs or alum were detected during the weekly weight monitoring (Figure S1). Additionally, we performed a weekly, comprehensive assessment of the health status of the mice from the different groups as previously described [32]. These assessments showed no relevant abnormalities. For the six representative mice immunized three times with the highest dose (40 µg) of the mix of PreS-RBD and the three non-treated mice, histopathological examinations of organs (i.e., liver, spleen, kidney, lung and hearts) were performed. As exemplified by the representative animals, no significant differences between immunized and non-treated groups were detected as observed via necropsy and histological evaluation (Figure S2).

### 3.3. Immunization with W-PreS-O Induces High IgG Antibody Levels Specific for Wild-Type and Omicron RBD

**Figure 3:**
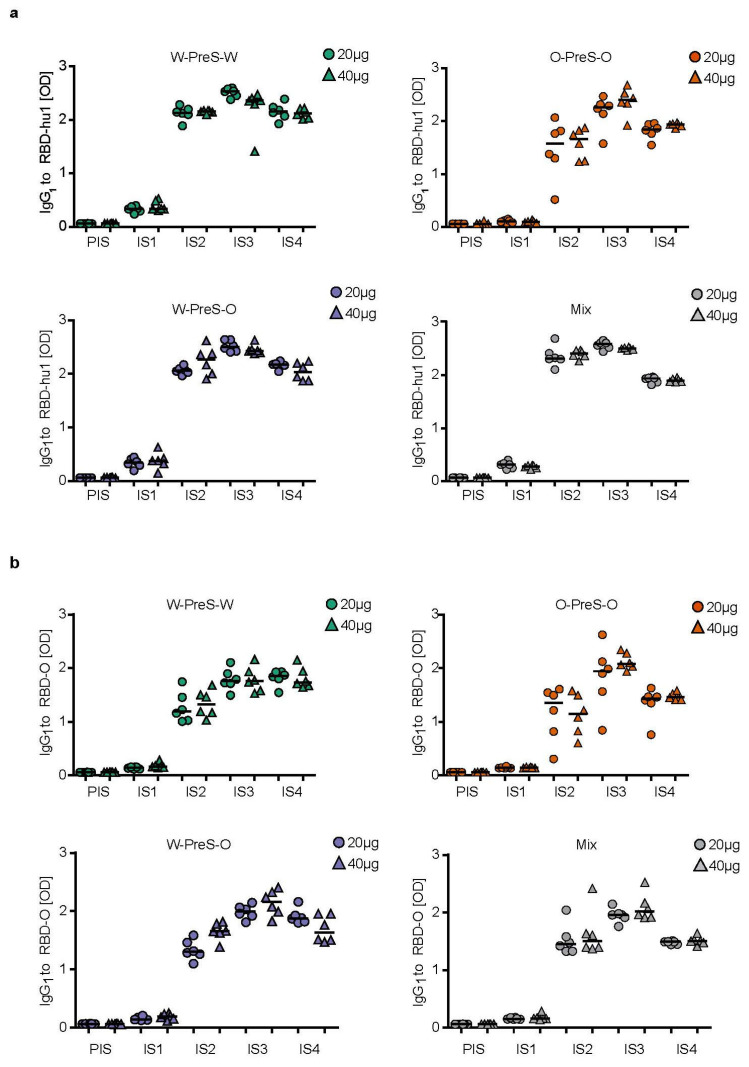
Time course of RBD-specific IgG_1_ responses in immunized mice. (**a**) RBD-hu-1- and (**b**) RBD-Omicron-specific IgG_1_ responses are shown in serum samples from mice (1:500 dilution), immunized with 20 µg (circles) or 40 µg (triangles) of PreS-RBD fusion protein vaccines at indicated time points (x-axes). OD_405/492 nm_ values corresponding to IgG_1_ antibody levels are shown (y-axes). Horizontal bars represent median values for each group.

Figure 3 shows the time-dependent kinetics of the IgG_1_ antibody levels specific for wild-type RBD (Figure 3a) and Omicron RBD (Figure 3b) in mice immunized with different doses of the different vaccines. The first RBD-specific antibodies became detectable already three weeks after the first immunization, and antibody levels strongly increased three weeks after the second and third immunization (Figure 3). For each of the tested vaccines (W-PreS-W, O-PreS-O, W-PreS-O, mix of W-PreS-W and O-PreS-O), Omicron RBD-specific IgG_1_ levels were somewhat lower than wild-type RBD-specific antibody levels at the corresponding time points, but these differences were not statistically significant (Figure 3a,b). The vaccines, including wild-type RBD, induced higher wild-type RBD-specific IgG_1_ levels than only the Omicron RBD-based vaccine (Figure 3a). Again, this difference was not significant.

However, the trend that vaccines containing strain-specific RBDs induced higher IgG_1_ levels to the corresponding RBD was not observed for Omicron (Figure 3b). W-PreS-W, the mix groups and especially W-PreS-O induced robust Omicron-specific IgG_1_ levels which were comparable or even higher than Omicron RBD-specific IgG_1_ levels induced with O-PreS-O. Of note, lower levels of Omicron RBD-specific IgG_1_ levels in the O-PreS-O (median OD IgG_1_ IS3 20 µg: 1.94; 40 µg: 2,07; IS4 20 µg: 1.43; 40 µg: 1.46) and in the mix groups (median OD IgG_1_: IS3 = 20 µg: 1.95; 40 µg: 2,01; IS4 = 20 µg: 1.48; 40 µg: 1.50), compared to the W-PreS-W (median OD IgG_1_: IS3 = 20 µg: 1.75; 40 µg: 1.75; IS4 = 20 µg: 1.84; 40 µg: 1.73) and W-PreS-O (median OD IgG_1_: IS3 = 20 µg: 1.99; 40 µg: 2.15; IS4 = 20 µg: 1.87; 40 µg: 1.63) groups were observed (Figure 3a). However, these results were also not significantly different from each other. Furthermore, we noted that RBD-specific IgG_1_ levels induced with O-PreS-O in the individual mice were more heterogeneous (i.e., results were more scattered with a higher standard deviation) than those induced with the other vaccines. With the exception of W-PreS-W-induced Omicron-specific IgG_1_ levels, we observed a small but distinct decline in RBD-specific IgG_1_ levels at time-point IS4 (i.e., six weeks after the last immunization) (Figure 3a,b).

### 3.4. The Aluminum-Hydroxide Adsorbed W-PreS-O Vaccine Induces a Mixed Th1/Th2 Response Specific for RBD

**Figure 4:**
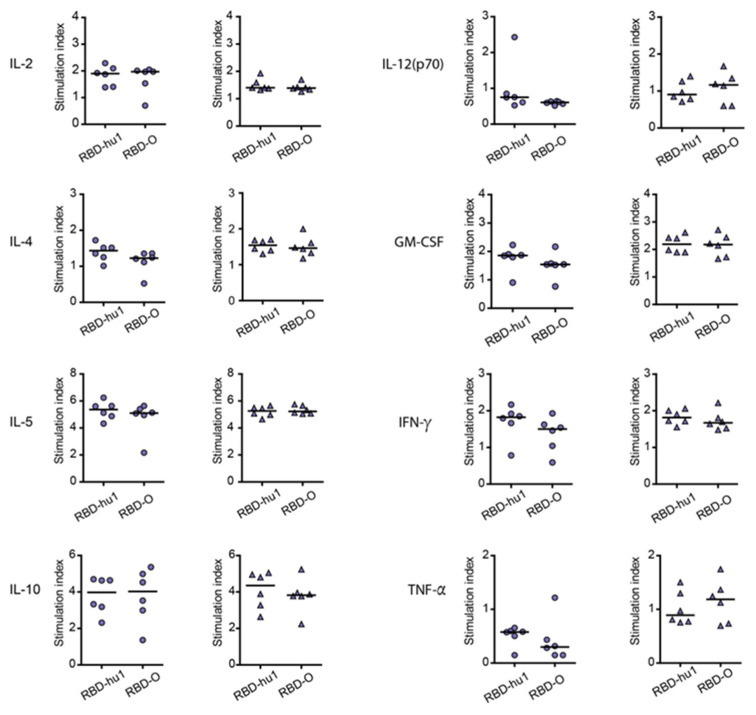
Secretion of RBD-specific cytokines of supernatants from cultured splenocytes of mice immunized with 20 μg (circles) or 40 μg (triangles) of W-PreS-O, after stimulation with RBD-hu-1, RBD-Omicron or medium. The stimulation indices of cytokines are shown (y-axes; IL-2, IL-4, IL-5, IL-10, IL-12, GM-CSF, IFN-γ, TNF-α) specific for wild-type RBD (RBD-hu-1) or Omicron RBD (RBD-O) (x-axes), calculated as the average measurement of cytokines divided by the average of triplicate of unstimulated cells. Horizontal bars represent median values of groups.

The cytokine responses to wild-type RBD and Omicron RBD were measured in supernatants of cultured splenocytes obtained at time-point IS4 from mice immunized with the different PreS-RBD vaccines. Figure 4 shows the RBD-specific production of cytokines in mice immunized with 20 µg or 40 µg W-PreS-O. We found comparable effects of the PreS-RBD vaccines on cytokine responses for both doses of vaccines and for RBD from wild-type and Omicron SARS-CoV-2 (Figure 4). An induction of RBD-specific Th2 cytokines (i.e., IL-4, IL-5) but also RBD-specific Th1 cytokines (IFN-γ, GM-CSF) and the tolerogenic cytokine IL-10 was observed. An increase in IL-2 indicative of T cell stimulation was noted, whereas the inflammatory cytokines (TNF-α, IL-12) were even found to be slightly decreased. A balanced Th1/Th2 ratio (IFN-γ/IL-4) of 0.91 for RBD-hu-1 and 0.88 for RBD-Omicron was noted, suggesting that the alumn-adjuvanted PreS-RBD-based vaccine induced a balanced RBD-specific Th1/Th2 response.

**Figure 5:**
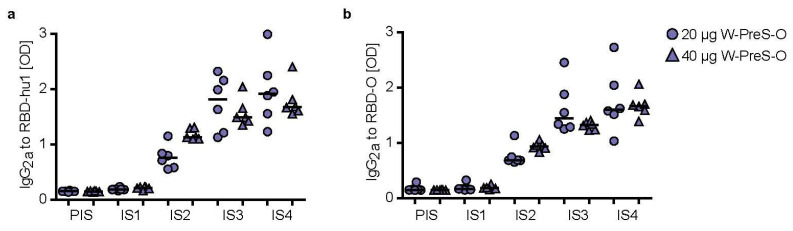
IgG_2a_ levels specific for (**a**) RBD-hu-1 and (**b**) RBD-Omicron measured in 1:500 diluted serum samples of mice immunized with 20 µg (circles) or 40 µg (triangles) of the W-PreS-O vaccine at indicated time-points (x-axes). OD_405/492nm_ values corresponding to specific antibody levels (y-axes) are shown as the average of duplicate determinations for individual animals (*n* = 6 per group) with <5% deviation. Horizontal bars represent median antibody levels for each group.

In the murine system, the balanced production of specific IgG_1_ and IgG_2a_ antibodies is indicative of a mixed Th1/Th2 immune response. Therefore, we also measured RBD-specific IgG_2a_ levels in sera from immunized mice, using the very same serum dilution which had been used for measuring specific IgG_1_ levels (Figure 5). Figure 5 shows the kinetics and levels of IgG_2a_ toward wild-type (Figure 5a) and Omicron RBD (Figure 5b) in mice immunized with 20 μg or 40 μg of W-PreS-O. A robust induction of IgG_2a_ specific for RBD from both SARS-CoV-2 strains was observed with specific levels of IgG_2a_ corresponding to RBD-specific IgG_1_ levels (Figure 3 and Figure 5). There was no statistically significant difference regarding the induction of RBD-specific IgG_1_ and IgG_2a_ antibody levels for the two doses (i.e., 20 μg or 40 μg of W-PreS-O). However, we noted a difference regarding the kinetics of antibody production when comparing the development and duration of RBD-specific IgG_1_ and IgG_2a_ antibodies. The RBD-specific IgG_1_ increased earlier than the RBD-specific IgG_2a_ (Figure 3 and Figure 5). Importantly, the RBD-specific IgG2_a_ levels did not show a decrease at time-point IS4 when compared to IS3, whereas IgG_1_ showed a decrease (Figure 3 and Figure 5). In detail, the following results were obtained: for 20 µg median OD hu-1-sIgG2a IS3: 1.81, IS4: 1.91; OD Omicron-sIgG_2a_ IS3: 1.44, IS4: 1.56 and for 40 µg (OD hu-1-sIgG2a IS3: 1.48, IS4: 1.67; OD Omicron-sIgG_2a_ IS3: 1.32, IS4: 1.67) doses from three weeks (IS3) to six weeks after the third immunization (IS4) (Figure 5a,b).

### 3.5. W-PreS-O Induced the Highest Virus Neutralization Titers against Omicron 

**Figure 6:**
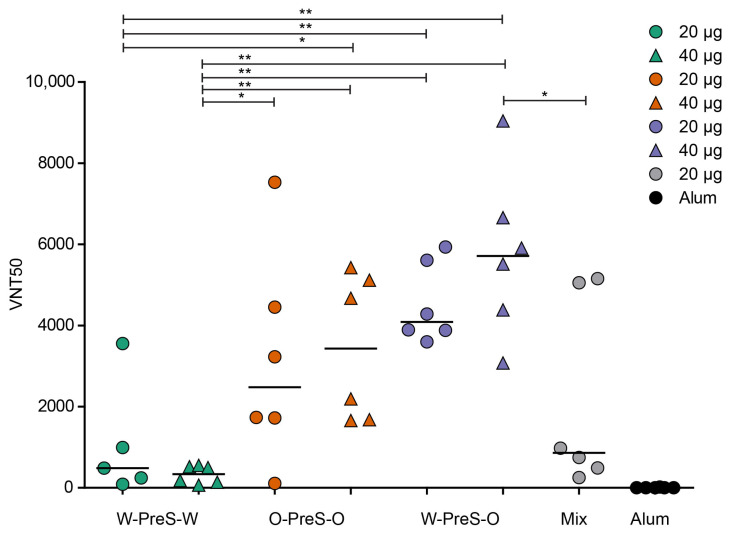
SARS-CoV-2 Omicron neutralization via antibodies induced with the PreS-RBD vaccines. VNT50 titers (y-axis) obtained with serum samples (IS4) of mice immunized with PreS-RBD fusion proteins or alum alone as indicated (x-axis) are shown as median values of triplicate determinations for each serum. Median VNT50 titers are shown with horizontal bars. Significant differences between each of the PreS-RBD-vaccinated groups determined using a two-tailed Mann–Whitney U-test, *p* values: (** < 0.001, * < 0.01) are indicated.

It has been previously reported that high levels of RBD-specific IgG are correlated with high VNTs toward the SARS-CoV-2 wild-type strain [24,33] and that hu-1-induced antibodies partly cross-react with variants of concern [15,24,34]. However, functional assays, such as virus neutralization assays or molecular interaction assays, are especially useful for the measurement of antibodies protecting against virus infection [12,35,36]. Therefore, serum samples obtained from mice six weeks after the third immunization with the PreS-RBD fusion constructs were analyzed regarding their capacity to neutralize SARS-CoV-2 Omicron (Figure 6).

We found that the W-PreS-O-induced antibodies had the highest SARS-CoV-2 Omicron virus neutralization titers (20 µg VNT50 min: 3595, max: 4938, median: 4088; 40 µg VNT50 min: 3076, max: 9042, median: 5711). In contrast, the virus-neutralizing capacity of the W-PreS-W-induced antibodies was modest (20 µg VNT50 min: 87, max: 3554, median: 482; 40 µg VNT50 min: 66, max: 552, median: 338) compared to W-PreS-O and O-PreS-O (Figure 6). Interestingly, immunization with the bivalent mix comprising the two strain-specific antigens, W-PreS-W and O-PreS-O, led to much lower VNT50 titers (20 µg mix VNT50: min: 254, max: 5155, median: 860) than the corresponding W-PreS-O vaccine (i.e., 20µg) (median VNT50: 4088). However, even more interesting was the fact that the median virus neutralization titers against Omicron induced with the O-PreS-O vaccine were considerably lower (20 µg VNT50 min: 109, max: 7530, median: 2480; 40 µg VNT50 min: 1658, max: 5429, median: 3430) than those induced with the W-PreS-O vaccine (20 µg VNT50 median: 4088; 40 µg VNT50 median: 5711). However, these differences were not statistically significant.

### 3.6. Lack of Association of W-PreS-O-Induced Omicron-Specific Antibody Levels with Neutralization of Omicron 

When analyzing the kinetics of RBD Omicron-specific IgG_1_ and IgG_2a_ in mice immunized with W-PreS-O, we found different kinetics of antibody responses. Omicron-specific IgG_1_ increased and declined earlier than Omicron-specific IgG_2a_ (Figure 3 and Figure 5). In order to obtain information regarding a possible association of RBD Omicron-specific IgG_1_ and IgG_2a_ responses, and thus between the synchronization of subclass responses and/or epitope specificity, we performed a correlation analysis of both subclass responses (Figure S4). There was a weak correlation between RBD wild-type-specific IgG_1_ and IgG_2a_ antibody levels at time-points IS2 and IS3, which disappeared completely at time-point IS4 (Figure S4a). No correlation between RBD Omicron-specific IgG_1_ and IgG_2a_ antibody levels was found at any of the three time-points, i.e., IS2, IS3 and especially not at time-point IS4 when Omicron-specific virus neutralization was studied (Figure 5).

Next, we investigated if there was any association between RBD Omicron-specific IgG_1_ or IgG_2a_ levels induced with immunization with W-PreS-O at time-point IS4. In fact, Figure S5 shows that there was no correlation of RBD wild-type- or RBD Omicron-specific IgG_1_ levels with Omicron-specific virus neutralization titers even when tested with different serum dilutions ranging from 1:500 to 1:8000 (Figure S5). Likewise, we studied at time-point IS4 if RBD Omicron-specific IgG_2a_ levels induced with immunization with W-PreS-O correlated with Omicron-specific virus neutralization titers. However, no correlation of the antibody levels and virus neutralization titers was observed either for the mice which had been immunized with 20 μg or those immunized with 40 µg (Figure S6). No correlation was found even when the mice immunized with either 20 μg or 40 μg were analyzed together (Figure S6).

## 4. Discussion

Although Omicron and Omicron subvariants have accounted for the majority of SARS-CoV-2 infections for the last two years, only relatively few Omicron-specific SARS-CoV-2 vaccines are currently available (covid19.trackvaccines.org (accessed on 1 December 2023)). We have previously reported the construction, in vitro and in vivo characterization of a SARS-CoV-2 subunit vaccine, which is based on a recombinant fusion protein consisting of HBV-derived PreS and two SARS-CoV-2 wild-type RBDs, which are termed PreS-RBD [25]. The recombinant PreS-RBD fusion protein could be produced via expression in HEK cells as a soluble, folded protein in large amounts. HBV-derived PreS was included into PreS-RBD as an immunological carrier protein to ensure additional PreS-derived T cell help [37]. Accordingly, we observed that all rabbits immunized with PreS-RBD developed RBD-specific neutralizing antibodies. This could not be achieved with a vaccine based only on RBD without a carrier protein, indicating that the PreS-RBD vaccine may indeed overcome immunological non-responsiveness due to the integration of PreS as the immunological carrier.

The aim of this study was to improve the previously described PreS-RBD vaccine regarding the induction of Omicron-neutralizing antibodies. For this purpose, we constructed a fusion protein which was identical to the previous PreS-RBD (W-PreS-W) except that Wuhan RBD was replaced with Omicron RBD (O-PreS-O), and another construct which contained one Wuhan RBD and one Omicron RBD (W-PreS-O). We were able to express and purify all three vaccine antigens (i.e., W-PreS-W, O-PreS-O, W-PreS-O) as soluble and folded proteins and easily formulate them via adsorption to aluminum hydroxide. The three vaccines based on single recombinant fusion proteins were then compared to each other and to an equimolar mix of W-PreS-W and O-PreS-O (i.e., a bivalent vaccine) regarding their abilities to induce wild-type RBD- and Omicron RBD-specific antibodies. All four vaccines were well tolerated by the vaccinated mice as indicated with the clinical symptom scores, gain in body weight and histopathological examination, suggesting that they have a favorable safety profile.

Furthermore, all tested vaccines induced comparable IgG_1_ antibody responses that were specific for wild-type RBD. Omicron RBD-specific IgG_1_ antibody responses were slightly higher in the W-PreS-O immunized mice compared to the mice immunized with W-PreS-W, O-PreS-O or the bivalent mix of W-PreS-W and O-PreS-O, but the differences were not statistically significant. The levels of Omicron RBD-specific IgG_1_ antibodies were comparable to those of Omicron RBD-specific IgG_2a_ levels. Thus, it seemed that the aluminum hydroxide-adsorbed vaccines induced a mixed Th1/Th2 phenotype. This was confirmed through analysis of the cytokine responses, which were detected in supernatants of cultured splenocytes obtained from the immunized mice after stimulation with wild-type and Omicron RBD. Specific cytokine responses upon stimulation with RBD-hu-1 and RBD-Omicron were studied in mice immunized with W-PreS-O but not in mice immunized with W-PreS-W and O-PreS-O because immunization with W-PreS-O had induced the best virus-neutralizing antibody responses for Omicron.

In fact, we found a parallel and equivalent induction of Th2 cytokines (i.e., IL-4 and IL-5) as well as of Th1 cytokines (IFN-γ, GM-CSF), whereas there was a slight decrease in inflammatory cytokines (TNF-α, IL-12). In fact, as shown previously, certain vaccines can induce slight decreases in inflammatory cytokine levels without affecting the specific antibody responses [38,39,40]. Thus, the aluminum hydroxide-formulated vaccines seemed to induce a balanced immune response.

Regarding antibody responses, we found a profound difference regarding the kinetics of the RBD-specific IgG_1_ and IgG_2a_ responses. RBD-specific IgG_1_ levels increased more quickly than RBD-specific IgG_2a_ but decreased much earlier than RBD-specific IgG_2a_ levels.

Thus, it seems that specific IgG_1_ responses dominate in the early phase of protection, whereas IgG_2a_ antibodies are more relevant for sustained protection, at least in the murine model. One possible, but not exclusive, explanation for the different kinetics of specific IgG_1_ and IgG_2a_ responses may be that a sequential class switch has occurred in the immunized mice, because in mice, the Cγ1 constant region gene is located within the *Igh* locus upstream of the Cγ2a gene but downstream of the Cμ gene [41]. Therefore, we investigated whether there is a correlation of the levels of RBD-specific IgG_1_ and IgG_2a_. However, there was no significant correlation between RBD-specific IgG_1_ and IgG_2a_ levels, and it is therefore possible that both a sequential but also a direct class switch to IgG_1_ and IgG_2a_, most likely to a varying degree, has occurred in the individual animals. Alternatively, it is possible that the IgG_1_ and IgG_2_ subclass responses originate from different B cell clones following an independent class-switch program involving a Th1 and Th2 pathway. The latter possibility would be in accordance with either the lack of and/or the poor correlation of RBD-specific antibodies.

The most important finding of our study came from the assessment of the Omicron-neutralizing antibody titers. Although there were only minor differences regarding RBD-specific IgG_1_ and IgG_2a_ antibody levels, we found that the W-PreS-O-induced antibodies had the highest SARS-CoV-2 Omicron virus-neutralization capacities, which exceeded by far those induced with the W-PreS-W-based vaccine. The W-PreS-O-neutralizing antibody titers were also higher than those induced with the bivalent W-PreS-W and O-PreS-O mix and, surprisingly, also higher than the Omicron-specific VNTs induced by the O-PreS-O-based vaccine. The finding that the median VNT induced with the W-PreS-O-based vaccine was approximately twice as high as that of the O-PreS-O vaccine may be explained by the fact that the W-PreS-O-based vaccine is able to activate a broader repertoire of RBD-specific T cells and B cells, resulting in a broader T cell and antibody response. The findings that VNT titers did not depend only on specific antibody levels and that there was stronger scattering of VNT titers than that of antibody levels may be explained by the fact that virus neutralization does not only depend on the specific antibody levels but also on the fine specificities and avidities of to their epitopes.

Future studies will now focus on evaluating if vaccination with W-PreS-O can induce antibodies which prevent infection and virus in propagation in in vivo infection models [42]. In particular, Syrian hamsters, which have been shown to mimic mild-to moderate COVID-19, will be suitable candidates for research [43]. Furthermore, extensive toxicological studies need to be completed before the vaccine can be evaluated in clinical studies with humans. In summary, so far, the available data suggest that W-PreS-O may represent a useful SARS-CoV-2 subunit vaccine suitable for repeated pre-seasonal booster immunizations to achieve sustained protection against a variety of SARS-CoV-2 strains and especially against the currently prevailing Omicron strains.

## 5. Conclusions

Our study indicates that the vaccine based on W-PreS-O is superior to the vaccines based on W-PreS-W, O-PreS-O and the bivalent mix of W-PreS-W and O-PreS-O regarding the induction of Omicron-neutralizing antibodies in a murine model. Furthermore, the W-PreS-O-based vaccine has the advantage of only one recombinant fusion protein being sufficient for the production of a vaccine conveying broadly neutralizing antibodies against strains related both to Wuhan and to Omicron.

## 6. Patents

P.G., B.K., W.F.P. and R.V. are the authors of a patent application regarding the vaccine.

## Data Availability

The datasets used and/or analyzed during the current study are available from the corresponding author on reasonable request.

## Supplementary Materials

The following supporting information can be downloaded at https://www.mdpi.com/article/10.3390/vaccines12030229/s1, Figure S1: Weight development of mice immunized with (a) alum alone (negative control) or with 20 µg or 40 µg of the PreS-RBD fusion proteins (b–e) as measured weekly (x-axes) as gram body weight (y-axes); Figure S2: Representative hematoxylin–eosin stains of liver, spleen, kidney, heart and lung sections obtained from a non-immunized mouse (upper panel) or a mouse immunized with 40 µg of the mixture of W-PreS-W and O-PreS-O (high dose of PreS-RBD, lower panel); Figure S3: IgG_1_ levels specific for (a) RBD-hu-1 and (b) RBD-Omicron of mice immunized with 20µg (circles) or 40µg (triangles) of the PreS-RBD fusion proteins; Figure S4: Association of IgG_1_ and IgG_2a_ antibody levels specific for (a) RBD-hu-1 and (b) RBD-Omicron of mice immunized with 20 µg or 40 µg of W-PreS-O at time-points IS2, IS3 and IS4; Figure S5: Lack of correlation of virus neutralization titers (VNT50, x-axes) in sera of mice (IS4) immunized with W-PreS-O (W-O) with levels of IgG antibodies (OD values, y-axis) to folded RBD-hu-1 (left) and RBD-Omicron (right) measured at dilutions (a) 1:500, (b) 1:1000, (c) 1:2000, (d) 1:4000 and (e) 1:8000; Figure S6: Lack of correlation of virus neutralization titers (VNT50, x-axes) in sera of mice (IS4) immunized with W-PreS-O with levels of IgG2a antibodies (OD values, y-axis) to folded Omicron RBD measured at dilutions 1:500 of (a) both groups, (b) mice immunized with 20 µg and (c) mice immunized with 20µg W-PreS-O.
