# Supplementary material for: Vaccine Based on Recombinant Fusion Protein Combining Hepatitis B Virus PreS with SARS-CoV-2 Wild-Type- and Omicron-Derived Receptor Binding Domain Strongly Induces Omicron-Neutralizing Antibodies in a Murine Model
Source: Vaccines (Basel). 2024 Feb 23;12(3):229. doi: 10.3390/vaccines12030229 (PMC10975884; doi:10.3390/vaccines12030229)
Supplement: Supplementary file 1 [file vaccines-12-00229-s001.zip › vaccines-2846429-supplementary.pdf]

Supplemental figures

Vaccine based on recombinant fusion protein combining HBV PreS with SARS-CoV-2 wild-type- and Omicron-derived RBD strongly induces Omicron-neutralizing antibodies

Pia Gattinger [1], Bernhard Kratzer [2], Al Nasar Ahmed Sehgal [2], Anna Ohradanova-Repic [3], Laura Gebetsberger [3], Gabor Tajti [3], Margarete Focke-Tejkl [1,4], Mirjam Schaar [1], Verena Fuhrmann [1], Lukas Petrowitsch [5], Walter Keller [5], Sandra Högler [6], Hannes Stockinger [3], Winfried F. Pickl [2] and Rudolf Valenta [1,4,7,8*]

[1] Medical University of Vienna, Center for Pathophysiology, Infectiology and Immunology, Department of Pathophysiology and Allergy Research, Division of Immunopathology, Vienna, Austria;
[2] Medical University of Vienna, Center for Pathophysiology, Infectiology and Immunology, Institute of Immunology, Vienna, Austria;
[3] Medical University of Vienna, Center for Pathophysiology, Infectiology and Immunology, Institute for Hygiene and Applied Immunology, Vienna, Austria;
[4] Karl Landsteiner University of Health Sciences, Krems, Austria;
[5] University of Graz, BioTechMed Graz, Institute of Molecular Biosciences, Graz, Austria;
[6] University of Veterinary Medicine Vienna, Institute of Pathology, Unit of Laboratory Animal Pathology, Vienna, Austria;
[7] Sechenov First Moscow State Medical University, Department of Clinical Immunology and Allergology, Laboratory for Immunopathology, Moscow, Russia
[8] NRC Institute of Immunology FMBA of Russia, Moscow, Russia

* Correspondence: rudolf.valenta@meduniwien.ac.at

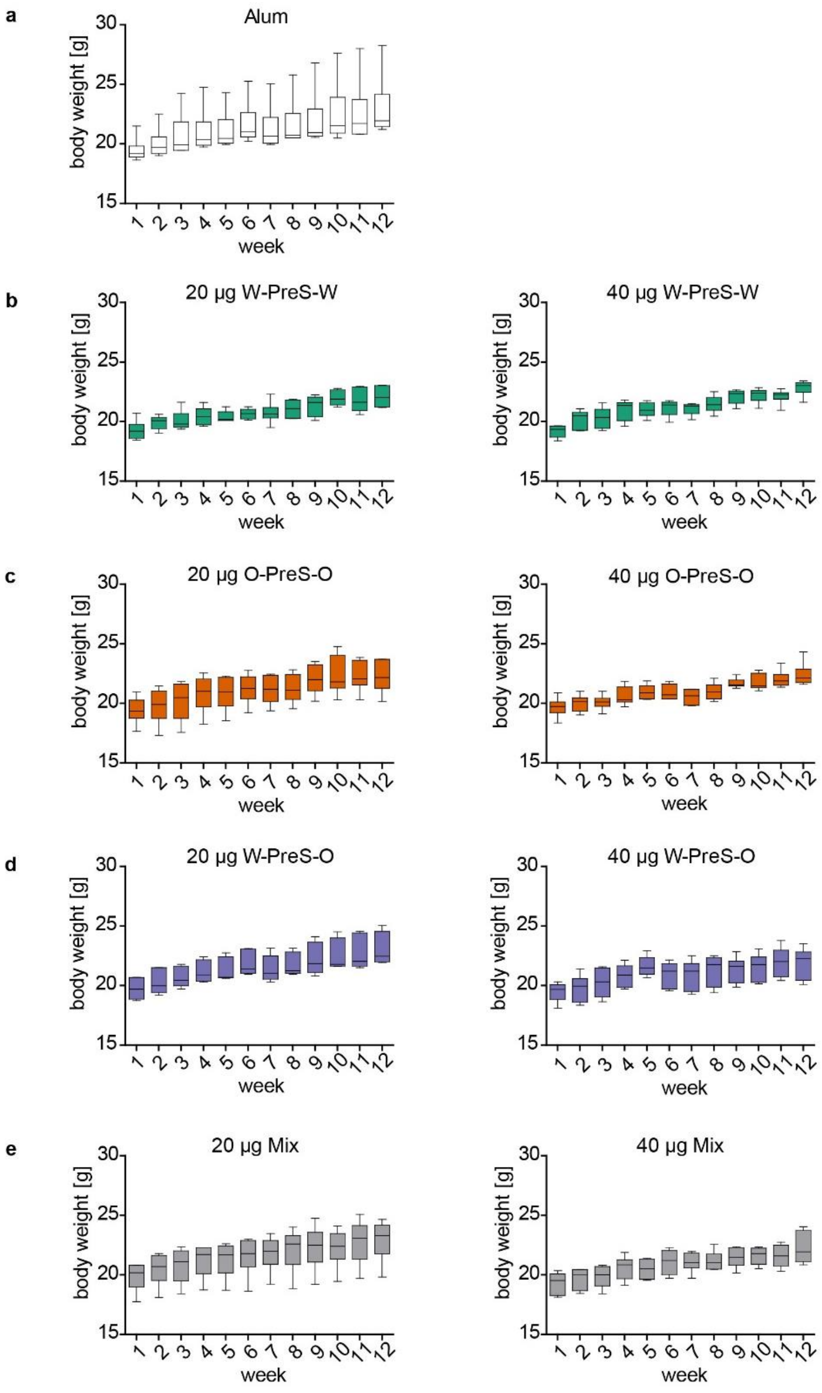

Figure S1. Weight development of mice immunized with **(a)** alum alone (negative control) or with 20 µg or 40 µg of the PreS-RBD fusion proteins **(b-e)** as measured weekly (x-axes) as gram body weight (y-axes). Shown are box-plots for each group (n=6 mice per group) with minimum and maximum values and medians indicated.

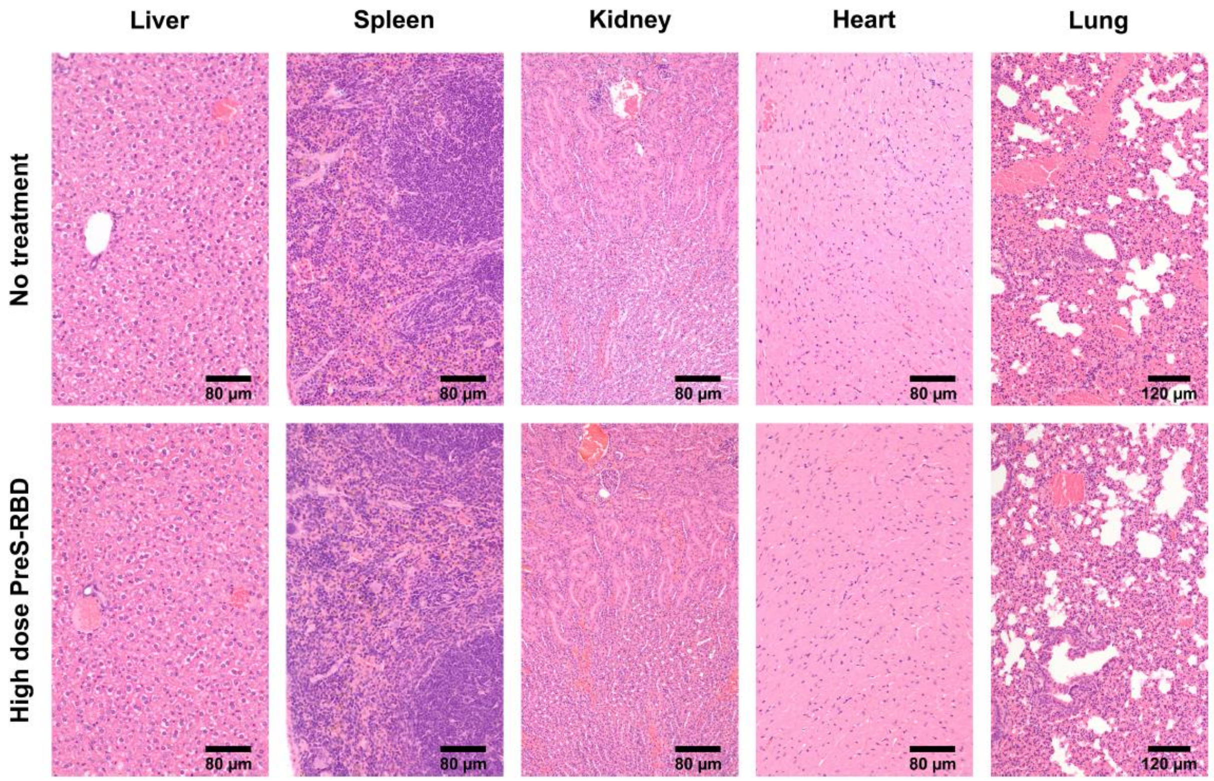

Figure S2. Representative hematoxylin–eosin stains of liver, spleen, kidney, heart and lung sections obtained from a non-immunized mouse (upper panel) or a mouse immunized with 40 µg of the mixture of W-PreS-W and O-PreS-O (high dose of PreS-RBD, lower panel).

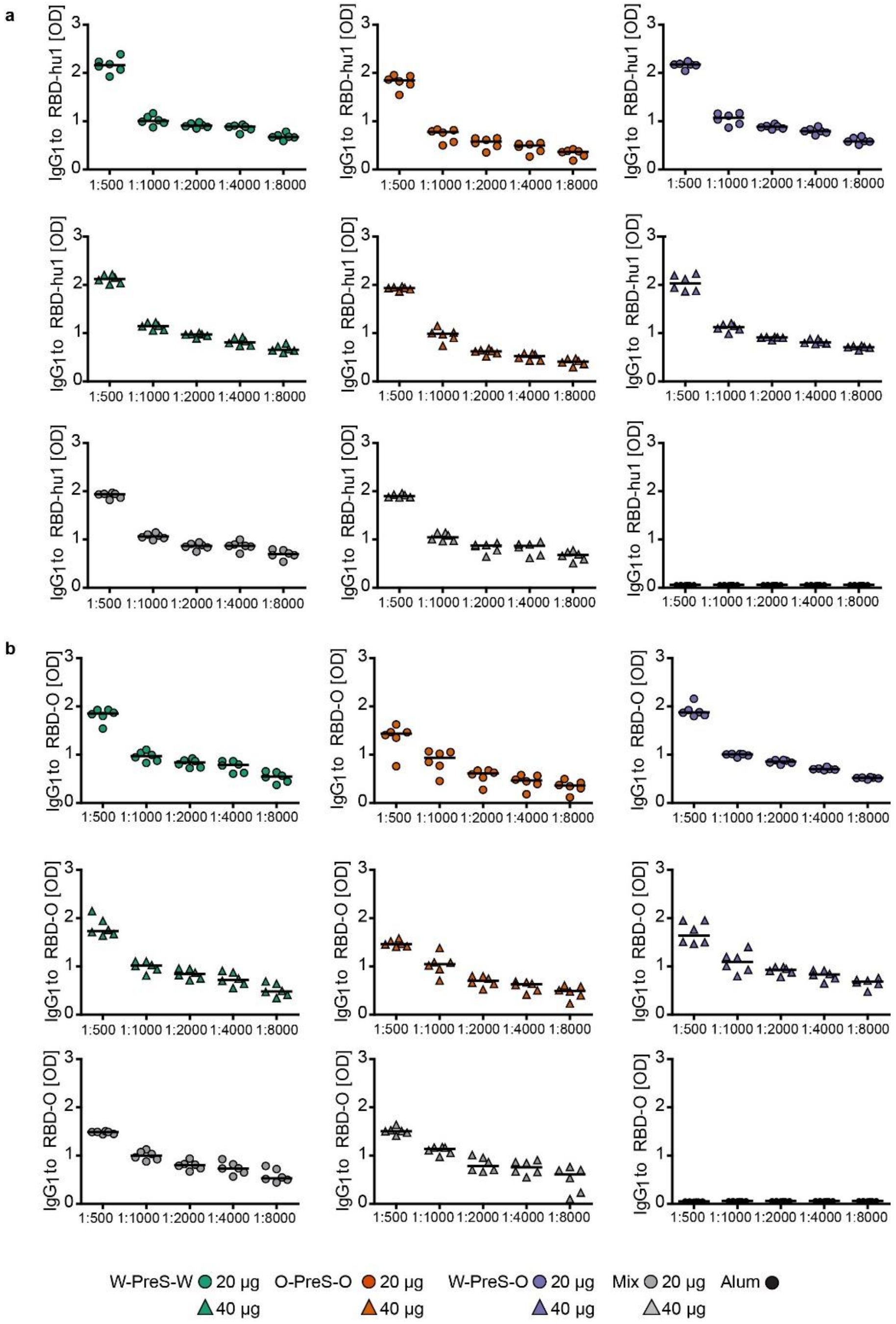

Figure S3. IgG1 levels specific for **(a**) RBD-hu1 and **(b)** RBD-Omicron of mice immunized with 20 µg (circles) or 40 µg (triangles) of the PreS-RBD fusion proteins as indicated below. Specific IgG1 levels were measured at indicated dilutions of serum samples (x-axes) and $OD_{405/492\ nm}$ values corresponding to IgG1 levels are shown as duplicate determinations for each animal (n= 6 per group) with <5% deviation. Horizontal bars denote median antibody levels.

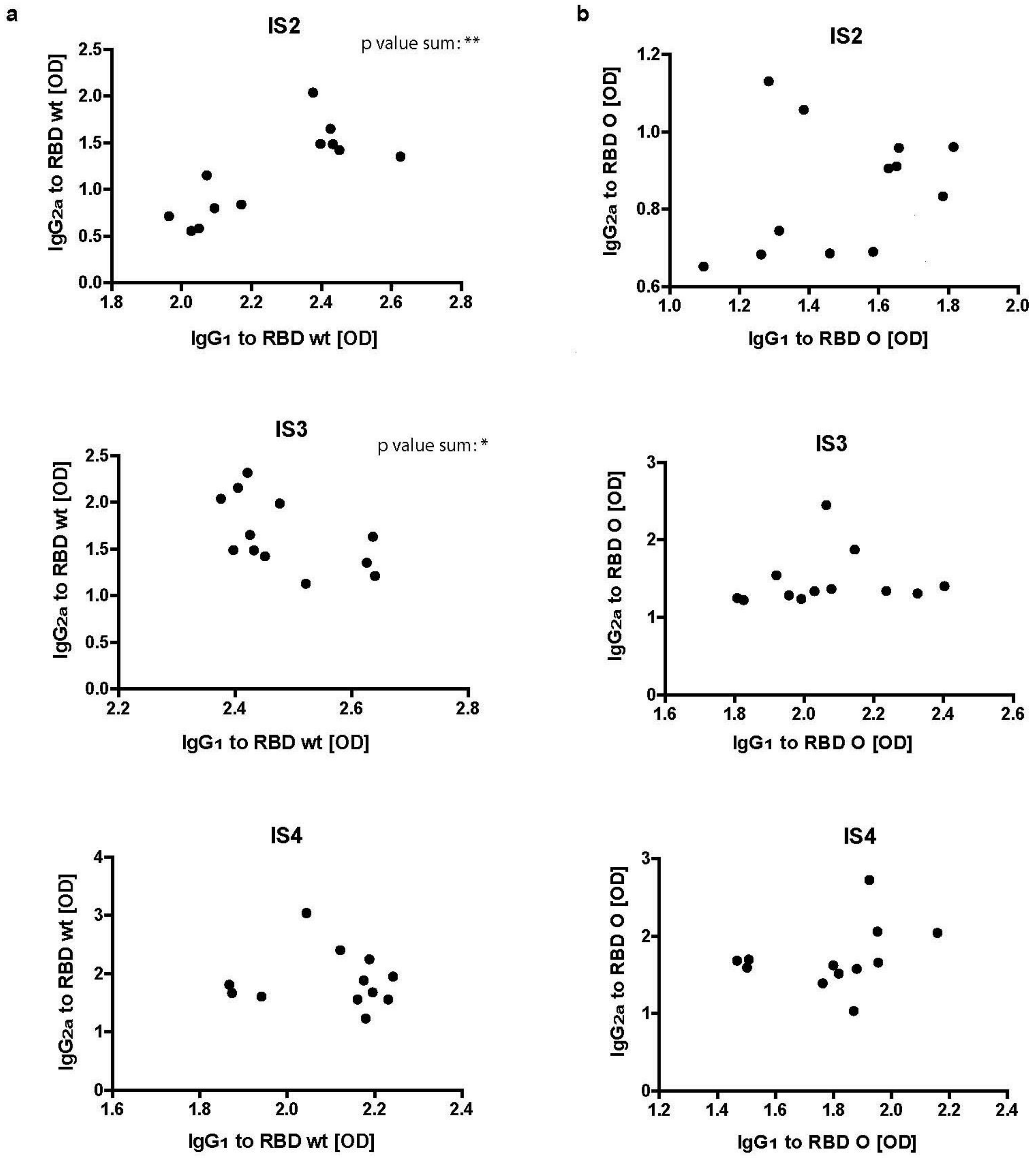

Figure S4. Correlation of IgG_1 and IgG_{2a} antibody levels specific for **(a)** RBD-hu1 and **(b)** RBD-Omicron of mice immunized with W-PreS-O at time points IS2, IS3 and IS4. Nonparametric Spearman´s correlation p values of <0.05 ($* < 0.05$, $** < 0.01$) were considered as significant.

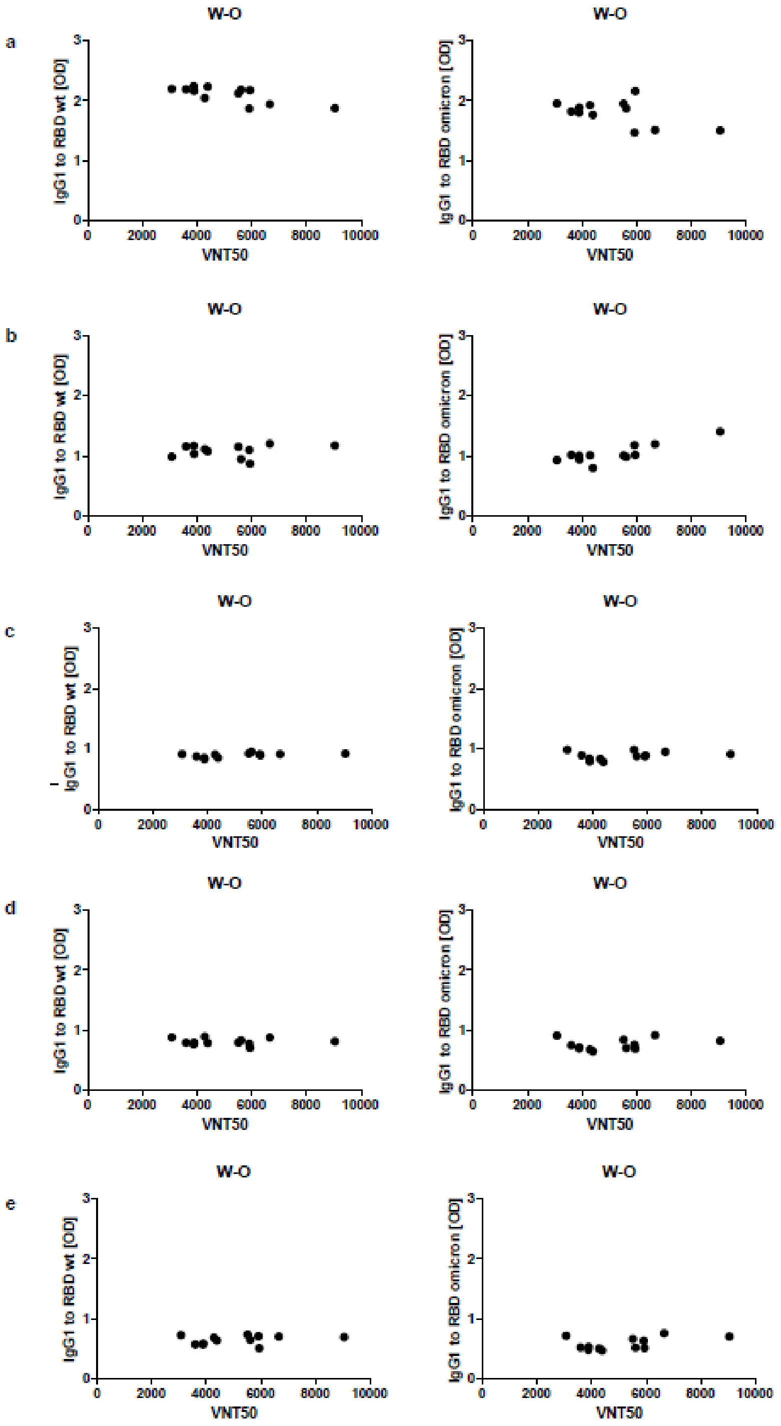

Figure S5. Lack of correlation of virus neutralization titers (VNT50, x-axes) in sera of mice (IS4) immunized with W-PreS-O (W-O) with levels of IgG_1 antibodies (OD values, y-axis) to folded RBD-hu1 (left) and RBD-Omicron (right) measured at dilutions **(a)** 1:500, (**b)** 1:1000, (**c)** 1:2000, (**d)** 1:4000 and **(e)**1:8000.

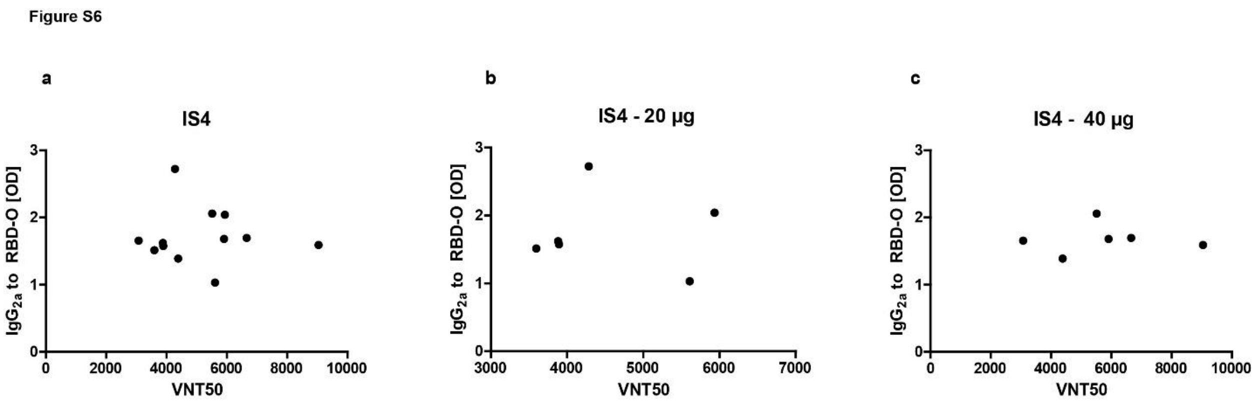

Figure S6. Lack of correlation of virus neutralization titers (VNT50, x-axes) in sera of mice (IS4) immunized with W-PreS-O with levels of IgG antibodies (OD values, y-axis) to folded RBD-Omicron measured at dilutions 1:500 of **(a)** both (20 µg and 40 µg) groups combined, **(b)** mice immunized with 20 µg and **(c)** mice immunized with 40 µg W-PreS-O.
